# Manchester Triage System: Effectiveness, Limitations, and Global Implementation—A Structured Narrative Review

**DOI:** 10.3390/healthcare14162502

**Published:** 2026-08-12

**Authors:** Jerica Zaloznik Djordjevic, Zvonka Fekonja, Matej Strnad

**Affiliations:** 1 Department of Emergency Medicine, University Medical Centre Maribor, 2000 Maribor, Slovenia; 2 Faculty of Health Sciences, University of Maribor, 2000 Maribor, Slovenia; 3 Prehospital Unit, Dr. Adolf Drolc Community Health Centre Maribor, 2000 Maribor, Slovenia; 4 Faculty of Medicine, Department of Emergency Medicine, University of Maribor, 2000 Maribor, Slovenia

**Keywords:** Manchester Triage System, emergency department triage, inter-rater variability, point-of-care testing

## Abstract

Background: The Manchester Triage System (MTS) is one of the most widely used emergency department (ED) triage tools worldwide, aiming to standardize patient prioritization through structured flowcharts and discriminators. Despite its broad adoption, concerns remain regarding its reliability, adaptability, and performance across diverse clinical and operational contexts. Objective: This narrative review aimed to identify and narratively synthesize evidence on the validity, reliability, and contextual performance of the MTS, with particular attention to inter-rater variability, cultural and linguistic adaptation, and operational limitations in real-world ED settings. Methods: A structured literature search was conducted across four databases from inception to January 2026 and analyzed according to the methodological recommendations for narrative biomedical reviews proposed by Gasparyan et al. to identify studies assessing the performance, implementation, and limitations of the MTS across different healthcare systems. Included studies were analyzed qualitatively, focusing on outcome measures related to accuracy, consistency, and contextual applicability. Results: The reviewed evidence supports the MTS’s ability to identify high-acuity patients; however, considerable variability in reliability and performance was observed across settings. Subjectivity in symptom interpretation and discriminator selection, challenges in non-English-speaking environments, and the lack of integration of operational factors, such as ED crowding and staffing levels, were recurrent findings. Conclusions: While the MTS remains a cornerstone of emergency triage, its effectiveness is influenced by human, cultural, and system-level factors. Future research should explore complementary strategies, including enhanced training, local adaptation, point-of-care testing, and artificial intelligence-based decision support, to improve triage consistency and patient outcomes.

## 1. Introduction

The primary purpose of triage is to ensure that patients receive timely medical care based on clinical urgency rather than arrival time in accordance with available medical and nursing resources [1]. The Manchester Triage System (MTS) was developed in the United Kingdom in the 1990s [1], following clinicians’ recognition that triage was chaotic. A group of around 20 senior emergency physicians and nurses from different emergency departments (EDs) developed a solution to be used in all EDs in the city of Manchester. Although it was not envisaged that the MTS would extend beyond the city of Manchester, it was first published in 1996 and later used in at least 90% of the UK’s EDs. Nowadays, it is one of the most widely used triage systems in emergency departments (EDs) across Europe and worldwide [2]. MTS assigns patients to one of five categories, ranging from immediate to non-urgent, based on presenting symptoms and predefined flowcharts [3].

Since its inception, the system has been adapted and implemented in diverse clinical settings, with varying degrees of success. The effectiveness, accuracy, and reliability of MTS have been the subject of considerable research [4,5]. This review critically evaluates current literature on the MTS, focusing on its clinical performance, validation studies, limitations, and recent adaptations in global emergency care settings.

## 2. Methods

### 2.1. Review Design

This study was conducted as a structured critical narrative review with an interpretive thematic synthesis as per Sukhera [6]. The purpose was to provide a broad, critical, and interpretive synthesis of evidence on the MTS, including its clinical performance, reliability, implementation challenges, contextual limitations, and future directions. A structured narrative review was considered the most appropriate design because the objective was to synthesize heterogeneous evidence on the validity, reliability, implementation, contextual adaptation, and operational aspects of the MTS rather than to produce pooled quantitative estimates of diagnostic accuracy.

The review was guided by the following question: How valid, reliable, and clinically effective is the MTS across different patient populations, emergency care settings, and healthcare systems, and which human, cultural, and operational factors influence its performance? The review was primarily intended for emergency physicians, triage nurses, researchers, educators, and healthcare decision-makers involved in implementing and evaluating emergency department triage systems.

### 2.2. Search Strategy and Data Sources

A structured literature search was performed using search terms related to the MTS and emergency department triage. Search MeSH terms included combinations of keywords such as “Manchester Triage System,” “MTS,” “emergency department triage,” “triage validity,” “triage reliability,” “overtriage,” “undertriage,” and “emergency care” using Boolean operators (AND, OR). The operator OR was used to combine synonymous or related terms, whereas AND was used to combine different concepts to narrow the search. Following the initial literature search, additional targeted searches were conducted as the analysis progressed and new themes emerged.

The literature search was conducted using the PubMed, CINAHL, Cochrane Library and Web of Science databases. PubMed and the Cochrane Library were selected to identify relevant medical literature and evidence syntheses, CINAHL to capture nursing and triage-focused research, and Web of Science to provide broader interdisciplinary and citation coverage. No publication year restriction was applied because both important early studies concerning the development and validation of the MTS and recent studies addressing the implementation were relevant to the review question. The final search was conducted on 23 January 2026. Only articles published in English were included. The reference lists of selected studies were also screened to identify additional relevant publications.

### 2.3. Eligibility and Study Selection

Studies were included if they evaluated the clinical performance, reliability, validity, implementation, adaptations, or comparative use of the MTS in emergency care settings. Comparative studies involving other triage systems, including the Australasian Triage Scale (ATS), Canadian Triage and Acuity Scale (CTAS), Emergency Severity Index (ESI), and South African Triage Scale (SATS), were also considered when relevant to the objectives of the review.

Studies were excluded if they were unrelated to emergency department triage, did not specifically address the MTS, lacked sufficient methodological or clinical detail, or consisted of editorials, conference abstracts without full text, or non-English publications.

Titles and abstracts were reviewed by the first author and subsequently approved by two senior co-authors for relevance to the objectives of this narrative review. Full texts were then examined when articles appeared to address the clinical performance, implementation, adaptation, or limitations of the MTS. The final selection was based on eligibility criteria, including relevance to the objectives of the review, evaluation of the MTS, and adequate methodological and clinical detail. To ensure broad representation, studies from different patient populations, clinical settings, and healthcare systems were included whenever they met the eligibility criteria. The included publications were analyzed inductively. Initial codes were generated from the findings of the selected literature, and related codes were subsequently grouped into broader themes through iterative review and discussion. The selection process was not intended to provide an exhaustive or reproducible evidence map of all available studies.

As this was a narrative review in accordance with the article by Gasparyan et al. [7] and the Scale for the Assessment of Narrative Review Articles (SANRA) principles [8], no formal protocol registration, duplicate screening, standardized risk-of-bias assessment, or meta-analysis was performed.

### 2.4. Data Extraction and Thematic Analysis

The analysis of the research incorporated into this review was performed in two consecutive phases. Initially, we synthesized and compared the methodological and contextual attributes of the identified papers. Secondly, we conducted thematic analysis following the methodology established by Braun and Clarke [9], which involved familiarization with the data, generation of initial codes, and review of themes. Themes were then defined, refined, and finalized through iterative discussion and consensus among the research team.

Study selection, coding, thematic development, and interpretation involved the researcher’s judgement. The authors’ professional background in emergency medicine, nursing, healthcare education, and research helped identify and interpret clinically relevant themes, particularly those concerning inter-rater variability, vulnerable patient groups, and workflow challenges. To reduce the influence of an individual perspective, themes were reviewed, discussed, and agreed upon by the authors.

The final thematic framework was considered sufficient when the selected literature covered the main clinical, methodological, and implementation areas relevant to the review objective. This indicated sufficient thematic coverage within the selected evidence, rather than complete coverage of all literature on the MTS. The first author performed the initial coding and grouped related codes into broader themes. The thematic structure was subsequently reviewed and discussed by the two senior co-authors. However, independent duplicate coding and formal assessment of inter-coder agreement were not performed.

### 2.5. Methodological Considerations

This review was conducted as a structured critical narrative synthesis. Its quality was supported by a clear review question, a transparent search and selection process, inclusion of key relevant studies, structured comparison of study characteristics, thematic analysis, team discussion, and acknowledgement of researcher judgement. The review was not intended to include every publication of MTS performance. The findings should therefore be understood as a critical and context-based synthesis rather than a complete or quantitatively weighted assessment of MTS validity or effectiveness.

## 3. Results

The structured search identified 90 potentially relevant records. After removal of duplicates and a relevance-based assessment of titles, abstracts and full texts, 27 publications were retained as the core evidence base for this narrative synthesis, including 20 original research studies and 7 review articles. Review articles were not treated as primary empirical evidence but were used to contextualize findings from original studies and to identify broader methodological and conceptual trends. When a review article summarized a primary study already included in the analysis, the reported findings were not treated as separate or additional evidence.

### 3.1. Characteristics of the Included Studies

In total, 27 publications were included (Table S1), encompassing a wide range of research designs, clinical populations, and healthcare settings, reflecting the diversity of approaches used to evaluate MTS. A condensed summary of the included studies is presented in Table 1, with studies grouped according to their clinical theme or presenting symptoms (Table 1).

Regarding research design, the included studies were predominantly quantitative and observational. The most frequently employed design was the retrospective cohort study, particularly in investigations assessing the validity and clinical performance of the MTS in specific symptom-based or disease-specific patient groups, such as shortness of breath, chest pain, headache, sepsis, gastrointestinal bleeding, and transient loss of consciousness [13,14,19,20,21,22,23,24,25,31,32]. A smaller proportion of studies adopted prospective observational designs, allowing for real-time data collection and more accurate assessment of clinical outcomes [11,17,18,26,27,30]. In addition to primary empirical studies, the review included several systematic reviews and narrative review articles that addressed broader issues of triage validity, reliability, and conceptual foundations [5,10,12,15,28,29,35]. Studies using simulated or hypothetical clinical scenarios were less common and were mainly used to evaluate inter-rater reliability [33].

In terms of geographical distribution, the majority of studies were conducted in Europe, particularly in countries where the MTS is well established in routine emergency care, including the Netherlands [11,26,30,31], Germany [17,25,32], Italy [5,14,20,21,22,24], Portugal [10,19], Spain [23,34], Poland [13,35] and the United Kingdom [18]. Additional studies from Australia [16,33], Brazil [10,28,29], Japan [15], and Thailand [27] provide insight into the application and adaptation of the MTS across different healthcare systems and socioeconomic contexts. Most studies were undertaken in high-income healthcare settings, with a smaller number conducted in low- and middle-income countries, where the MTS was often compared with locally developed triage tools.

Regarding conceptual frameworks, the included studies most commonly addressed the MTS in relation to triage urgency, clinical severity, predictive validity, reliability, and patient safety. Urgency was rarely defined using a direct gold standard. Because no universally accepted gold standard exists for MTS effectiveness, the review considered several complementary outcome definitions, including hospital admission [10,11,12,15,19,27,31], intensive care admission [11,14], resource utilization [11,15,31], and short-term mortality [10,11,12,14,15,19,20,30,31]. These outcomes were not treated as equivalent but were interpreted in each study.

Only a limited number of studies assessed urgency using more direct clinical indicators, such as the need for immediate life-saving interventions [17]. In pediatric [27,28,29] and geriatric [30,31] populations, additional concepts such as age-adjusted vital signs, frailty, and vulnerability were incorporated into the analytical frameworks.

The measurement approaches used across studies were predominantly quantitative, relying on routinely collected clinical and administrative data from emergency department information systems. The primary measurement instrument was the MTS, evaluated as an independent tool or compared with other triage systems, including the Emergency Severity Index (ESI) [12,13,15,27,28,29], Canadian Triage and Acuity Scale (CTAS) [12,15,27,28,29], Australasian Triage Scale (ATS) [12,15,27,29,33], and South African Triage Scale (SATS) [12]. Several studies also examined the MTS in combination with complementary scoring systems, such as the National Early Warning Score (NEWS) [14], the HEART score [19], or geriatric screening instruments (e.g., Acutely Presenting Older Patient (APOP)) [30], particularly in disease-specific or high-risk populations. Qualitative approaches were rare and were largely restricted to narrative or conceptual discussions within review articles.

The sample sizes varied considerably across studies, ranging from small single-centre cohorts of approximately 150–500 patients [18,19,21,32,34] to large-scale observational studies including more than 100,000 patients [26], and, in some cases, over 280,000 emergency department visits [11]. Most primary studies were conducted in single-centre settings, which may limit the generalisability of findings. However, several large prospective or registry-based studies incorporated multiple emergency departments or healthcare institutions. Sampling strategies most commonly involved consecutive patient inclusion or analysis of all eligible cases within a defined time period, while reliability and simulation studies frequently used purposive or convenience sampling of healthcare professionals.

Overall, the included studies constitute a methodologically heterogeneous but thematically coherent body of evidence, offering a broad perspective on the performance, limitations, and contextual influences affecting the use of the MTS across diverse clinical populations and emergency care settings.

### 3.2. Results of Data Synthesis

The thematic analysis of conceptual and methodological issues that emerged in the primary research and review articles yielded seven predominant themes, shown below: (1) background and development of MTS, (2) core structure and triage flowcharts, (3) validation and reliability studies, (4) clinical effectiveness in practice, (5) international adaptations, (6) critiques and limitations, and (7) comparison with alternative systems. Overall, these themes demonstrate that MTS performance is influenced by three overarching factors: (a) variability in clinical interpretation, (b) patient- and population-specific characteristics, and (c) healthcare system and operational context. These findings provide a framework for understanding both the strengths and limitations of MTS in real-world practice.

The publication category, role in the manuscript, relevant section, and inclusion in the inductive analysis for all cited references are presented in Supplementary Table S2.

#### 3.2.1. Background and Development of MTS

Early triage systems originated from the need to classify surgical patients in battlefield environments, with the principles of patient prioritization and on-site care emerging in France in the early nineteenth century. These systems subsequently evolved, largely within military contexts, during the conflicts of the nineteenth and twentieth centuries. As organized medical systems developed in the Western world, triage began to be implemented in emergency departments in the United States, the United Kingdom, and elsewhere across Europe in the early twentieth century. At that time, triage typically involved a rapid clinical assessment to determine the priority [36].

MTS was developed in response to growing concerns about inconsistencies and inefficiencies in patient prioritization within EDs in the United Kingdom. Prior to the introduction of structured triage protocols, triage decisions were often informal, subjective, and varied widely between clinicians and institutions [37,38]. This inconsistency had direct implications for patient safety and timely access to care [3].

The absence of a risk classification system in ED leads to patient management based on order of arrival, which in many cases may compromise patient safety, as individuals with more unstable or severe health conditions are not prioritized in a timely manner [39].

MTS was introduced as a standardized method for assessing the urgency of patients’ conditions based on presenting complaints, clinical discriminators, and vital signs. It operates using a set of symptom-based flowcharts, each leading to one of five triage categories: Red (Immediate), Orange (Very Urgent), Yellow (Urgent), Green (Standard), or Blue (Non-urgent). Each category is associated with a target maximum waiting time, ranging from 0 min (Red) to 240 min (Blue). A guiding principle in its design was to ensure that patients with life-threatening conditions were reliably identified and prioritized while maintaining workflow efficiency [3].

Over time, the MTS has undergone various refinements, with updates incorporating new clinical knowledge, pediatric-specific flowcharts, and improvements to enhance usability across different healthcare settings. MTS has seen widespread adoption internationally, often with local adaptations to reflect regional epidemiological and healthcare system differences [4,26,40,41].

#### 3.2.2. Core Structure and Triage Flowcharts

At the heart of MTS lies a structured framework that standardizes patient assessment based on presenting complaints. The system uses 53 symptom-based flowcharts, each guiding the triage nurse through a logical decision-making pathway to assign the patient to one of five urgency categories. Each flowchart begins with a general presenting complaint, such as chest pain, shortness of breath, or abdominal symptoms, and guides the triage nurse through a series of clinical “discriminators.” Discriminators include both objective criteria (e.g., respiratory rate, oxygen saturation, level of consciousness) and subjective indicators (e.g., pain severity). They are arranged in the ABCDE format and can be general, meaning they apply to all patients regardless of their presentation (e.g., acuity, life threat), or specific, applying to individual presentations and relating to key features of those presentations (e.g., cardiac pain, pleuritic pain, aortic pain). The presence or absence of these discriminators determines the urgency category assigned [3,31].

Flowcharts are organized by symptom group and are supported by a manual that defines each discriminator in detail, reducing ambiguity in interpretation [3]. Some flowcharts also have specialized pathways for pediatric patients, who often present differently from adults and may not be able to articulate symptoms as clearly [26].

Importantly, the MTS is designed to minimize the risk of under-triage. This means that when any high-risk discriminator is present, the system defaults to a higher urgency category. While this design principle enhances patient safety, it also contributes to one of the system’s recognized limitations [42].

Despite its algorithmic appearance, the system allows some degree of clinical judgement. Triage nurses are trained to override the assigned category if a contextual or holistic assessment indicates a different level of urgency is warranted. However, such overrides must be justified and documented to ensure accountability and traceability [3].

#### 3.2.3. Validation and Reliability Studies

A prospective observational study by Zachariasse et al. [11], involving almost 300,000 patients, demonstrated moderate-to-good validity, with poorer performance in the most vulnerable patient populations: the young and the elderly. Moreover, the study reveals that it matters where you validate a triage system, as results are highly variable across clinical settings [11].

A retrospective, single-centre study evaluated 27,238 emergency department patients to compare the National Early Warning Score (NEWS) with the Manchester Triage System [14]. The primary outcomes were 30-day mortality, hospitalization, and ICU admission. Secondary outcomes were assessed in a random 5% subgroup and included the need for life-saving interventions, physician-defined clinical priority, and severity. NEWS showed superior accuracy for predicting 30-day mortality compared with MTS. In contrast, MTS significantly outperformed NEWS in predicting hospitalization, ICU admission, and all secondary, clinically relevant ED outcomes. The findings support existing evidence that mortality alone is a suboptimal endpoint for evaluating triage systems, as many post-triage factors influence it. Subgroup analyses reinforced that NEWS, while useful for mortality prediction, cannot replace structured triage systems. The study emphasizes improving and integrating existing triage frameworks rather than replacing them with early warning scores based solely on vital signs, which fail to capture the complexity of initial emergency department assessment.

A systematic review on validity by Kuriyama et al. [15] found that there were both construct (30-day mortality, ED length of stay or costs in EDs) (n = 10 trials) and criterion validity (immediate life-saving interventions or objective standard criteria/urgency set by expert panels) (n = 7 trials) studies found in the existing literature confirming MTS validation.

The prospective observational study by Gräff et al. [17] showed that, among patients requiring life-saving interventions, the MTS demonstrated a sensitivity of 75.0%, a negative predictive value (NPV) of 99.2%, a negative likelihood ratio (LR−) of 0.303, and an overall accuracy of 98.5% for triaging these patients into the red category. For patients requiring acute emergency treatment, appropriate classification into the red or orange triage categories showed a sensitivity of 82.0%, an NPV of 98.9%, a negative likelihood ratio (LR−) of 0.206, and an accuracy of 86.5%. MTS represents a valid and effective method for the initial assessment of critically ill emergency patients at presentation.

Predictive validity, referring to the system’s ability to accurately identify patients who require urgent care, has been more variable. In a systematic review and meta-analysis, Parenti et al. found that although MTS generally performed well at identifying high-acuity patients, its sensitivity was lower in specific subgroups, particularly elderly patients and those with atypical presentations [5].

The MTS showed poorer performance in older patients compared with younger individuals, as reflected by its reduced ability to predict in-hospital mortality in the older population. However, among older patients, the MTS performed more effectively in surgical than in medical cases, particularly with respect to hospital admission and in-hospital mortality [31]. One of the reasons for poorer performance is acute cognitive impairment, such as delirium or dementia. It is a high-risk factor for incorrect triage in geriatric patients as triage nurses often struggle to recognize it [43]. A secondary analysis of the APOP prospective study by Blomaard et al. [30] revealed that, within each triage urgency category, older patients (>70 years) with a high-risk geriatric screening result had a 3-fold higher 30-day mortality rate than patients identified as low risk during geriatric screening. Combining geriatric screening with triage urgency explained more of the variability of 30-day mortality in older ED patients than triage urgency alone. MTS alone had low discriminative performance for 30-day mortality in older ED patients with an AUC of 0.57; thus, combining triage urgency with geriatric screening has the potential to improve triage in this specific patient population [44].

On the other hand, MTS prioritizes safety in pediatric patients, resulting in substantially higher rates of over-triage than under-triage [29]. However, several studies reported that MTS showed unacceptably high hospital admission rates at lower levels of urgency, suggesting undertriage [21,22,23,24,25,28]. Accurately triaging patients with medical conditions, particularly younger children, remains especially challenging due to the need for specialized communication skills and different responses to physiological stressors (e.g., dehydration, infection) [45]. Validity was reduced in children presenting with medical conditions compared with those presenting with traumatic injuries [46].

A systematic review by Magalhães-Barbosa et al. [28] on the reliability of pediatric triage systems identified only one reliability study of the MTS conducted with actual pediatric patients. Van Veen et al. [47] included 198 consecutive patients at two Dutch centres who were independently triaged by both a triage nurse and a research nurse. Inter-rater reliability was substantial with moderate precision (κw = 0.65; 95% CI, 0.56–0.72). A systematic review on pediatric triage by Aeimchanbanjong et al. [27] found that MTS illustrated an AUC of 0.7 (95% CI, 0.66–0.74), sensitivity of 57% and specificity of 69%.

Several factors may explain the less consistent discriminatory performance of the MTS in older and pediatric populations. In older adults, acute illness may present with nonspecific symptoms, cognitive impairment, and attenuated physiological responses, which can make recognition of clinical urgency more difficult [30,31,43]. Furthermore, mortality and hospital admission in this population are influenced by frailty, comorbidity burden, and functional status, and may therefore be imperfect proxies for urgency at the time of triage [11,30,31]. In children, urgency assessment is complicated by age-dependent physiological ranges, limited symptom communication, nonspecific manifestations of illness, and differences between medical and traumatic presentations [26,27,28,29,45,46]. The performance of the MTS may also depend on the consistent application of age-adjusted vital signs in discriminators, with evidence suggesting that their incorporation can improve pediatric prioritization [26]. These factors may contribute to both safety-oriented overtriage and the under-recognition of serious illness in children with subtle or atypical presentations [27,28,29,46].

Inter-rater reliability has been a cornerstone of MTS validation. A simulation study conducted in the Netherlands confirmed moderate-to-substantial inter-rater reliability when flowcharts were applied consistently and with adequate training, and with high test–retest reliability. Its reliability was not influenced by nurses’ work experience [4].

Grouse et al. [16] retrospectively evaluated the inter-rater reliability of the MTS in an Australian emergency department using 50 triage scenarios derived from the notes of 50 consecutive patients who had presented to the emergency department. The Kappa value ranged from 0.4007 to 0.8018, with a median of 0.6304.

However, real-world conditions often yield lower reliability. Factors such as triage nurse experience, workload, patient volume, and ambiguous clinical presentations can lead to variability in category assignment. In a study comparing specially trained triage nurses with the MTS, 1270 patients were enrolled, and it was shown that nurses who undergo specialized triage training and continuous training demonstrate superior patient stratification and better risk prediction compared with reliance on the MTS alone [48].

Studies have shown that the inter-rater agreement drops significantly in high-pressure or pediatric environments, where symptoms may be less specific and interpretation of discriminators more subjective [5,49].

#### 3.2.4. Clinical Effectiveness in Practice

Effectiveness is typically measured by patient outcomes, time to clinical intervention, resource allocation, and ED workflow efficiency. The implementation of MTS was associated with a reduction in the time to treatment for high-priority cases, suggesting that the system functions effectively as an early-warning tool. This is particularly important in time-sensitive emergencies, where delays can significantly affect morbidity and mortality [39].

However, effectiveness in lower-acuity cases is less clear. Over-triage may result in patients with non-urgent conditions occupying fast-track pathways or urgent care treatment capacity, thereby contributing to ED overcrowding and prolonged waiting times for patients with truly urgent conditions [50].

In patients presenting with dyspnoea, MTS demonstrated good sensitivity for seven-day mortality when yellow, orange, or red categories were selected, effectively helping to avoid under-triage. However, the specificity of MTS for this non-specific symptom was low, indicating that a considerable proportion of patients with dyspnoea were over-triaged and consequently assigned a higher category despite having an acceptable lower priority [20]. Pulmonary embolism (PE) remains a significant clinical challenge for emergency care systems. A study that included patients with chest pain, cough, or collapse as the most frequent symptoms of PE showed that more than 55% of patients with clinically severe PE were triaged in the orange and red categories. While the MTS demonstrated good specificity and negative predictive values for PE in patients presenting with chest pain, dyspnoea, or collapse, its sensitivity was lower [21].

Chest pain is among the most frequent causes of ED visits and can be a sign of various life-threatening diseases. A retrospective single-centre study by Leite et al. [19] showed that fewer than 10% of patients presenting with chest pain had acute coronary syndrome (ACS). Although non-specific chest pain (mostly musculoskeletal pain) was the most common cause of chest pain, ACS was the second most frequent diagnosis. The MTS effectively triaged patients with ACS into the orange or red priority categories. However, delays in electrocardiogram (ECG) acquisition were observed (more than 10 min from patient arrival) in the green and yellow categories, which could potentially lead to significant delays in reperfusion therapy. Notably, no ST-elevation myocardial infarctions were identified among patients triaged as green or yellow. Overall, MTS demonstrated a reasonable discriminatory power. Similar results, demonstrating high sensitivity in assigning high priority (immediate or very urgent) to patients with ACS, were also reported in a Portuguese study [51]. It was also shown that nurses using the MTS can identify, with high sensitivity, which patients presenting to the emergency department with chest pain require early ECG recording and prompt physician assessment [18].

One of the most common neurological symptoms presenting in EDs is headache. The complexity of management stems from distinguishing between life-threatening and benign headache [52], for which the International Headache Society’s Secondary Headache Special Interest Group has defined 15 warning signs, also known as red flags, for detecting secondary headache [53]. The MTS does not include as many as 9 of these 15 warning signs (systemic symptoms, a history of neoplasm, age at onset older than 50 years, positional headache, headache triggered by coughing or Valsalva maneuvers, onset during pregnancy or the puerperium, eye pain, immunosuppression, and association with medication overuse); however, it does include items suggested in prospective studies or in clinical decision tools designed to assist in determining the indication for neuroimaging studies. In one study, MTS assigned most headache patients to the three intermediate triage categories, with similar distributions among patients with primary and secondary headaches. Importantly, undertriage was frequent: four out of five patients who met criteria for the very urgent category and one out of four patients who met criteria for the urgent category were undertriaged. These findings suggest that MTS has a limited ability to adequately identify and prioritize patients with secondary and high-risk secondary headaches [23]. In another study, MTS demonstrated good performance in safely excluding low-risk headache patients. Non-urgent (blue/green) priorities reliably ruled out severe cases, with a negative predictive value of 99.1%. However, the system struggled to identify all urgent cases and undertriaged 64.7% of patients who required urgent care, particularly those presenting with subjective symptoms or with symptom onset more than 24 h prior. The authors recommended the use of supplementary tools, such as symptom-based scoring systems, to improve the detection of time-sensitive pathologies [22].

Accurate characterization and clear prognostic definition are crucial in cases of transient loss of consciousness (TLOC), as it frequently presents with underlying acute and potentially serious medical conditions, with cardiac syncope as the most serious one [54]. In the analyzed population, cardiac syncope and epileptic seizures were more commonly observed in the high-priority group, and reflex syncope was more prevalent in the low-priority group, as well as severe acute disease- was observed at a higher percentage in the high-priority group (42.1% vs. 11.7%). MTS demonstrated low sensitivity but high specificity and relatively good accuracy in identifying severe acute disease; however, its discriminatory ability was only moderate, indicating that the system alone is insufficient to reliably exclude patients with severe acute disease [24].

During MTS validation in patients with gastrointestinal bleeding, it was found that MTS effectively categorized patients according to the presence of bleeding. Patients with a higher level of urgency had a higher proportion of confirmed gastrointestinal bleeding (67%), whereas patients with a lower triage category had confirmed bleeding less frequently (20%). Triage using the MTS in addition to established risk scores, such as the Glasgow-Baltchford score, could be the first step in a specific algorithm for a structured diagnostic and therapeutic approach, thereby minimizing the time lost until definitive treatment [32].

A retrospective observational study of 801 septic patients presenting to an ED in Bonn, Germany, showed that sensitivity was low in patients with sepsis and circulatory disorders (MTS sensitivity was 61.5% (95% CI 39.3 to 79.8) and LR was 0.466 (95% CI 0.286 to 0.757) [25]. The reason was the definition of the discriminator “shock”, which is described as sweating, pallor, tachycardia, and hypotension. It identifies patients with hemorrhagic shock, but in the context of systemic infection, the lack of cut-off blood pressure measurements may lead to inadequate triage. Therefore, they propose including blood pressure with a defined cut-off value in the MTS “orange” category, thereby improving the detection of patients with sepsis and circulatory dysfunction. In addition, the routinely assessed vital signs in the MTS should be recalibrated to align with the SIRS cut-off values, which are already incorporated into the NEWS system [14]. A specific algorithm for systemic infection is also suggested, recognizing that many elderly patients with systemic infection do not present with fever. Nonspecific symptoms such as chills, disorientation, apathy, loss of appetite, mottled skin and diarrhea should also be considered. At the same time, they suggest eliminating discrepancies between heart rate and body temperature cutoffs in MTS and the American College of Chest Physicians/Society of Critical Care Medicine Systemic Inflammatory Response Syndrome (ACCP/SCCM SIRS) criteria, extending blood pressure cutoffs to presentation flowcharts other than pregnancy, and including respiratory rate cutoffs [25]. Although a new definition of sepsis (sepsis 3) has been in effect since early 2016, and SIRS is no longer used as a criterion for the diagnosis of sepsis, indicators of organ dysfunction, such as changes in consciousness, respiratory rate, and blood pressure, are still used when sepsis is suspected [55].

Vital signs are incorporated into the MTS through individual discriminators and may therefore be measured selectively according to the presenting complaint, local protocol, or clinical judgement of the triage nurse. Consequently, early abnormalities in respiratory rate, blood pressure, oxygen saturation, temperature, or level of consciousness may not be consistently identified, particularly in patients with nonspecific or atypical presentations. This differs from physiological scoring systems such as the National Early Warning Score [14], which systematically assesses and combines several vital signs into a cumulative score. Such physiological systems may therefore be more sensitive to early deterioration or sepsis-related organ dysfunction, although they do not incorporate the same clinical context based on symptoms such as MTS.

#### 3.2.5. International Adaptations

Since its inception in the United Kingdom, the MTS has been adopted in numerous countries across Europe, South America, the Middle East, and parts of Asia. However, its global dissemination has not been a simple matter of translation; rather, it has required thoughtful local adaptation to accommodate variations in language, healthcare infrastructure, disease prevalence, patient demographics, and cultural norms [10,11].

MTS has been implemented and studied in several European countries, including the Netherlands, Germany, Portugal, Sweden, Spain, Austria and Slovenia [11,17,19,20,23,25,30,31,56]. In these settings, studies have generally found the system to be effective, particularly when implementation is accompanied by rigorous training and translation of triage flowcharts [57].

In Portuguese- and Spanish-speaking countries, MTS has undergone more extensive adaptations. In Spain, in 2010, the Spanish Triage System (SET) and MTS were the two most widely implemented standardized systems in EDs. MTS was chosen by the health services of Asturias, Galicia, Madrid, and the Valencian Community for their public hospitals [58]. In Brazil, where it is employed in both public and private hospitals, the system has been translated and culturally adjusted to reflect local clinical practices and epidemiology [49]. An adapted version of MTS can function effectively, but it also highlights persistent challenges, particularly in pediatric care and in settings with resource limitations or high-patient volumes [59].

#### 3.2.6. Critiques and Limitations

Although the literature search was structured to improve transparency and reduce selection bias, the review did not follow the full methodological requirements of a systematic review. In particular, no protocol was registered, no duplicate independent screening was performed, no standardized risk-of-bias assessment was undertaken, and no meta-analysis was planned or conducted. The findings should therefore be interpreted as a qualitative and thematic synthesis of the MTS’s validity or effectiveness.

One of the most frequently cited limitations is the risk of both under-triage and over-triage. Due to its conservative design, which defaults to higher urgency levels when any severe discriminator is present, the MTS often errs on the side of caution. While this approach enhances patient safety, it can lead to over-triage, especially in cases involving vague or nonspecific symptoms. Over-triage strains ED resources, contributes to overcrowding, and potentially delays care for patients who are genuinely in need of urgent intervention [10]. The balance between safety-driven over-triage and operational efficiency is inherently context-dependent. A certain degree of over-triage may be justified when it reduces the risk of delayed recognition and treatment of time-sensitive or life-threatening conditions [10,39]. However, excessive assignment of lower-risk patients to higher-priority pathways may increase the demand for urgent treatment areas, staff, and monitoring capacity. It may contribute to ED crowding and prolonged waiting times [50]. The consequences of over-triage should therefore be assessed alongside those of under-triage rather than considered in isolation. Evaluation of triage performance should include both patient-safety outcomes, such as missed critical illness and time to treatment, and operational outcomes, including waiting times, resource utilization, patient flow, and crowding [39]. The acceptable balance is likely to vary according to local patient population, staffing, capacity, and the clinical consequences of missed deterioration.

Conversely, under-triage is thought to be less common than over-triage and can occur in vulnerable groups such as older adults, children, and individuals with atypical presentations, leading to dangerous delays in care [5]. Under-triage mainly occurs in the MTS categories orange and yellow [4]. There is no agreement in the scientific literature on the exact prevalence of over- and undertriage among adult and child populations [5].

Another major critique centres on subjectivity and inter-rater variability, particularly in high-volume, high-stress settings [11,50,60]. Although MTS is intended to standardize decision-making through structured flowcharts and discriminators, studies have shown that symptom interpretation and discriminator selection can vary significantly among triage nurses [49]. This variability undermines the system’s reliability and highlights the need for continuous training and competency assessment [4].

The rigidity of flowchart pathways has also been criticized for limiting clinical judgment. While MTS allows triage nurse overrides, the process is not always encouraged or well-integrated into workflows. In fast-paced environments, the pressure to adhere strictly to algorithmic outputs may override a nurse’s intuition or clinical experience, particularly in ambiguous or complex cases [61].

In terms of cultural and linguistic adaptability, several studies conducted in non-English-speaking settings suggest that local adaptation of the Manchester Triage System may influence its performance and interpretation [10,19,27,34,62]. Even when translated, some clinical terms and symptom descriptions may lack direct equivalents, leading to misinterpretation and potential triage errors. Moreover, patient expectations about urgency and communication styles vary across cultures, complicating the triage process in multicultural or international contexts [49,59,61].

Lastly, MTS does not inherently account for operational factors, such as ED staffing levels, patient flow, or availability of downstream services [11,63]. It is designed as a clinical tool, yet in practice, triage decisions often intersect with logistical realities. This disconnection can limit the utility of the MTS as a comprehensive tool for managing ED throughput and performance.

#### 3.2.7. Comparison with Alternative Systems

Alternative triage systems are discussed by geographic origin and conceptual development, beginning with high-resource, five-level systems most comparable to the MTS, followed by systems specifically adapted for resource-limited settings.

While the MTS remains one of the most widely used triage tools in Europe and beyond, several alternative systems have been developed to meet the diverse demands of EDs worldwide [42,57].

Evidence suggests that five-level triage systems generally demonstrate greater discrimination and reliability than less differentiated three-level triage systems [15,57,64]. Among the most common five-level triage systems are the Manchester Triage System (MTS), the Emergency Severity Index (ESI), the Canadian Triage and Acuity Scale (CTAS), and the Australian Triage Scale (ATS) (Table 2).

The choice of tool should be guided by local needs, patient demographics, institutional resources, and staff expertise [65]. While MTS offers a robust framework for prioritizing clinical urgency, its effectiveness may be enhanced or limited by how well it is adapted and integrated into the broader context of emergency care delivery.

**Table 2:** Comparison of different triage systems.

Triage System	Core Principle	Reported Performance	Main Strengths	Main Limitations	Comparison with MTS
**Manchester Triage System (MTS)**	Symptom-based flowcharts with discriminators	Inter-rater reliability κ ≈ 0.40–0.80 (median ≈ 0.63) [4,16]; pediatric κw = 0.65 [47]; sensitivity 75% for life-saving interventions [17], 82% for acute emergency treatment [17]; older adults AUC 0.57 for hospital admission [30]; sensitivity 57% [27], specificity 69% (pediatric) [27]	Highly standardized; structured decision support; widely validated; effective for identifying high-acuity patients	Overtriage of nonspecific complaints; undertriage in elderly, pediatric and atypical presentations; does not account for resource needs or operational factors	Reference system
**Australasian Triage Scale (ATS)**	Clinical urgency based on the presenting condition [12]	Moderate validity and reliability in a pediatric review [29]	ATS demonstrated moderate validity [15,27]	In poisoning scenarios, it may assign higher acuity than MTS [33]; depends heavily on clinical judgement [12]	MTS may undertriage asymptomatic poisonings unless the “high-lethality overdose” discriminator is applied [33]
**Canadian Triage and Acuity Scale (CTAS)**	Presenting complaint with detailed clinical modifiers	High inter-rater reliability (pediatric κ ≈ 0.8–0.9) [28,29,42,66]	Comprehensive clinical modifiers; dedicated pediatric and obstetric pathways [27]; extensively validated [15,67]	More complex and potentially slower in high-throughput EDs [15]	MTS may be more practical where rapid triage and limited training resources are priorities [42]
**Emergency Severity Index (ESI)**	Acuity plus anticipated resource utilization	High predictive performance for pediatric hospital admission (AUC 0.78) [15]; good-to-high inter-rater reliability in pediatric studies [27,28], inter-rater reliability κ = 0.94 in a Spanish ED validation study [68]	Excellent prediction of admissions [13]; facilitates ED resource planning [64]; good reliability [38]	Greater reliance on clinical judgement; fewer pediatric modifiers [29]; risk of undertriage in older adults [15]	Outperforms MTS for pediatric admission prediction [27], whereas MTS provides more balanced patient distribution and greater standardization through flowcharts [38,69]
**South African Triage Scale (SATS)**	Presenting complaint + physiological early warning score	No superiority demonstrated over the other five-level systems	Well-suited for low-resource settings [12]; incorporates physiological scoring; practical for prehospital and rural care [70]	Less detailed symptom-specific guidance than MTS	Better suited for resource-limited environments, whereas MTS is preferable in tertiary centres with greater diagnostic capability [71]

## 4. Discussion and Future Directions

Early reviews by Parenti et al. [5] and Azeredo et al. [10] reported moderate-to-good validity and reliability of MTS, but highlighted substantial heterogeneity across studies and settings. Our findings align with these conclusions, confirming that MTS performs best for high-priority categories while showing less consistent discrimination among lower-acuity patients. Importantly, compared with these earlier reviews, our analysis incorporates more recent studies that further emphasize the influence of local implementation, staff training, and case-mix on observed performance. This suggests that the variability noted in earlier reviews has not been fully resolved over time, despite broader dissemination and longer experience with the system.

A recent review published in 2024 addressed similar aspects of the MTS [35]. While that review provided a comprehensive overview of existing evidence, the present narrative review differs in its structured thematic synthesis and its specific focus on operational and system-level factors influencing MTS performance. In addition, the inclusion of more recent studies allows further insights into emerging challenges and potential directions for refinement, particularly regarding implementation in diverse clinical settings [11,13,14,17,25,26,27,30,31].

Consistent with reviews of five-level triage systems more broadly, particularly those by Kuriyama et al. [15] and Hinson et al. [12], our review highlights persistent subjectivity in triage decision-making. Although MTS is designed to standardize assessment through flowcharts and discriminators, inter-rater variability remains a recurring issue. Our findings support prior observations that standardization alone cannot eliminate interpretive differences [15], especially in high-volume or resource-constrained EDs [45]. In this respect, our results do not differ substantially from previous reviews but rather reinforce the conclusion that human factors continue to play a central role in triage outcomes.

In pediatric settings, our findings are concordant with the reviews by Magalhães-Barbosa et al. [28] and Simon et al. [29], which reported variable reliability and limited evidence for the superior performance of any single triage system. Similar to these reviews, we found that MTS demonstrates acceptable reliability in some pediatric contexts but remains sensitive to age-specific presentation, staff experience, and local adaptation. Notably, our review further underscores the lack of robust comparative data in pediatric populations, suggesting that evidence gaps identified in earlier reviews persist.

A key contribution of our review, which extends beyond several earlier MTS-focused reviews, is the emphasis on operational and system-level factors. While previous reviews primarily assessed validity and reliability metrics, our findings demonstrate that appropriate triage categorization does not consistently translate into timely care when emergency department crowding, staffing shortages, or downstream bottlenecks are present. This aligns with broader critiques of triage performance raised by Hinson et al. [12], but contrasts with earlier MTS-specific reviews, which gave less attention to these contextual constraints. Our results, therefore, suggest that MTS performance should be interpreted within the wider organizational environment rather than evaluated as an isolated decision-making tool.

The international use of the MTS has required adaptation to local linguistic and healthcare contexts. In some settings, alternative triage systems have been developed or preferred to better align with regional clinical practice. This limitation, already noted by Parenti et al. [5] and Azeredo et al. [10], remains relevant and highlights ongoing challenges in the global transferability of MTS without rigorous local validation.

Future research should therefore move beyond algorithm-focused evaluations and explore strategies that integrate MTS within broader clinical and operational decision-support frameworks. Promising directions include incorporating point-of-care testing at triage to enhance early risk stratification [72,73]. Recent evidence suggests that artificial intelligence (AI) and machine-learning (ML) decisions may complement conventional triage systems by improving predictive discrimination, disease identification, and risk stratification. ML models have generally demonstrated greater discriminatory performance than traditional triage approaches and may assist in identifying patients requiring urgent hospitalization, predicting length of stay, and supporting more efficient allocation of ED resources. AI-assisted triage may therefore improve patient prioritization, reduce waiting times, and enhance the overall quality and efficiency of care. However, these potential benefits must be weighed against important implementation challenges, including variable data quality, algorithmic bias, limited clinician trust, ethical and transparency concerns, and uncertainty about generalizability across patient populations and healthcare systems. Prospective validation and careful integration into clinical workflows are therefore required before widespread adoption [74,75,76,77,78].

### Limitations of the Study

Several limitations of this review should be acknowledged. The limitations of the MTS can be considered at two distinct levels. First, internal flowchart limitations arise from the design of the system itself. These include reliance on symptom-based pathways, subjective interpretation of some discriminators, and the oversensitivity of selected high-risk discriminators, which may contribute to overtriage or undertriage [4,10,16,49,50,60]. Another structural limitation is that physiological parameters are not assessed consistently in all patients, which may be particularly important in those with nonspecific symptoms or early clinical deterioration [14,25].

Second, external implementation factors arise from the organizational context in which triage is performed. Differences in staff training and experience, staffing shortages, workload, high patient volume, emergency department crowding, limited monitored capacity, and restricted access to downstream services may prevent an appropriately assigned urgency category from advancing into timely assessment and treatment [11,12,39,48,63,65]. Internal limitations, therefore, primarily affect the accuracy and consistency of triage classification, whereas external factors influence the operational delivery of care after classification. Distinguishing between these levels is important because they require different interventions: refinement of flowcharts and discriminators for internal limitations, and workforce, workflow, and capacity measures for external implementation constraints.

An important methodological consideration is that this review was intentionally designed as a structured narrative review. Unlike a systematic review, its objective was not to generate pooled estimates of diagnostic accuracy, reliability, sensitivity, specificity, or predictive validity. Rather, the aim was to integrate heterogeneous evidence and provide a critical, thematic interpretation of recurring conceptual, clinical, and implementation-related issues. This approach enabled the inclusion of diverse sources, including primary empirical studies, systematic reviews, narrative reviews, and conceptual papers, thereby offering a broader and more context-sensitive understanding of how the MTS performs across different clinical and organizational settings.

A limitation of this narrative review is that no standardized risk-of-bias assessment was performed. Consequently, the methodological quality of the included studies was not formally evaluated using validated appraisal tools, and the findings should be interpreted with appropriate caution.

Inclusion and exclusion criteria were used to enhance transparency and reproducibility. Furthermore, the heterogeneity of study designs, patient populations, healthcare settings, and reported outcomes limited direct comparison between studies and precluded quantitative synthesis or meta-analysis. Consequently, the findings of this review should be interpreted as a qualitative narrative synthesis intended to provide an overview of current evidence on MTS, its applications, limitations, and emerging directions for refinement. As in previous reviews, heterogeneity in study designs, outcome measures, and reference standards limited direct comparisons, and publication bias cannot be excluded. In addition, the predominance of studies from high-income countries continues to restrict generalisability to low- and middle-income settings, an evidence gap repeatedly identified but not yet adequately addressed in the literature. Also, exclusion of non-English reports may limit the credibility of any statements about non-English settings.

Study screening and coding were performed by a single reviewer. Although two senior co-authors subsequently reviewed the final set of included publications, no independent duplicate screening or formal assessment of inter-reviewer agreement was conducted. This may have increased the risk of subjective selection decisions and of omitting potentially relevant studies.

## 5. Conclusions

This structured narrative review identified seven major themes related to the MTS, including its structural design, validation, clinical performance, and operational limitations. The reviewed evidence potentially suggests that MTS could remain a widely used and clinically useful framework for prioritizing patients according to urgency, particularly for identifying high-acuity cases. In particular, the synthesis highlights three overarching issues: (a) variability in reliability and validity across populations and settings, (b) the influence of subjective clinical interpretation on triage decisions, and (c) the limited integration of contextual and operational factors into the system. Together, these findings may have implications for clinical practice, system design, and future research. The MTS has been implemented in numerous EDs worldwide. It remains an important triage framework in contemporary emergency care, providing a structured, standardized approach to prioritizing patients based on clinical urgency. Some evidence included in this review indicates a potential association with identifying high-acuity patients, while also highlighting important limitations related to inter-rater variability, contextual dependence, and operational constraints. These findings suggest that triage performance may be shaped not only by algorithm design but also by local healthcare systems, workforce factors, and patient populations. Future efforts may explore optimizing MTS through targeted training, local adaptation, and integration with supportive technologies, such as point-of-care testing and artificial intelligence, with the aim of improving triage consistency and supporting patient care across diverse emergency department settings.

## Figures and Tables

**Table 1:** Condensed summary of the key included studies grouped by clinical theme.

Clinical Theme/Symptoms	Key Studies	Main Findings
**Overall validation of MTS**	Parenti 2014 [5]; Azeredo 2015 [10]; Zachariasse 2017 [11]; Hinson 2019 [12]; Ingielewicz 2025 [13]; Zaboli 2025 [14], Kuriyama 2017 [15], Grouse 2009 [16], Gräff 2019 [17]	MTS is a valid and reliable triage system for prioritization but demonstrates clinically relevant undertriage and overtriage in specific patient groups.
**Cardiac**	Speake 2003 [18]; Leite 2015 [19]	Good performance for chest pain prioritization, especially when combined with disease-specific tools such as the HEART score.
**Respiratory**	Ausserhofer 2020 [20]; Zaboli 2020 [21]	Good sensitivity for high-risk dyspnoea and unstable PE, but clinically stable PE patients may be undertriaged.
**Neurological**	Brigo 2022 [22]; García-Azorín 2023 [23]; Zaboli 2022 [24]	High negative predictive value but limited sensitivity for serious neurological conditions and syncope, leading to some undertriage.
**Sepsis**	Gräff 2017 [25]	Limited sensitivity for identifying sepsis, particularly severe sepsis and septic shock, resulting in substantial undertriage of critically ill patients.
**Pediatric**	Zachariasse 2021 [26]; Aeimchanbanjong 2017 [27]; Magalhães-Barbosa 2019 [28], Hany Simon 2022 [29]	Generally reliable, although younger children remain at risk of undertriage; adding age-specific vital-sign discriminators improves performance.
**Geriatric**	Blomaard 2020 [30]; Brouns 2019 [31]	Predictive accuracy decreases in older adults; combining MTS with frailty/geriatric screening improves risk stratification.
**Gastrointestinal**	Nguyen-Tat 2018 [32]	MTS showed high sensitivity but low specificity for identifying gastrointestinal bleeding cases.
**Special settings**	Jayaweera 2014 [33]; Castro Delgado 2022 [34]	MTS performs less well in toxicology and mass-casualty incidents compared with dedicated triage systems.
**Future directions**	Ingielewicz 2024 [35]	AI-assisted decision support and physiological parameters may improve future triage performance.

## Data Availability

No new data were created or analyzed in this study. Data sharing is not applicable to this article.

## Supplementary Materials

The following supporting information can be downloaded at: https://www.mdpi.com/article/10.3390/healthcare14162502/s1, Table S1. Characteristics of the included studies; Table S2. Role of all references used in the narrative review.
