# Supplementary material for: Manchester Triage System: Effectiveness, Limitations, and Global Implementation—A Structured Narrative Review
Source: Healthcare (Basel). 2026 Aug 12;14(16):2502. doi: 10.3390/healthcare14162502 (PMC13512649; doi:10.3390/healthcare14162502)
Supplement: Supplementary file 1 [file healthcare-14-02502-s001.zip › healthcare-4457068-Table S1.pdf]

Table S1: Characteristics of the included studies

Authors, year and country	Aim	Research design	Sample, setting	Main findings	Conceptual and methodological issues
Parenti et al. (2014), primarily in Italy with international collaboration (Italy–USA) (5).	To systematically review the international literature to evaluate the validity, reliability, and reporting quality of studies assessing the MTS in emergency departments.	Systematic review with narrative synthesis conducted according to PRISMA guidelines and methodological appraisal using STARD criteria.	Twelve observational studies (adult and paediatric ED populations) were conducted mainly in European emergency departments, particularly in the Netherlands and Scandinavia, using both real-patient data and triage scenarios.	The MTS demonstrates a wide range of inter-rater reliability (fair to very good) and generally good agreement, but shows low sensitivity, high rates of undertriage and overtriage (especially in children), and variable validity in predicting outcomes such as admission, workload, and mortality.	Evidence is limited by heavy reliance on scenario-based studies, inconsistent and proxy validity outcomes (e.g., mortality and admission), scarce intra-rater reliability data, heterogeneous reference standards, and variable methodological quality despite generally good reporting.
Jayaweera et al. (2014), Australia (33).	To compare whether the use of the ATS versus the MTS affects the urgency assigned to poisoned patients at the emergency department triage.	A multicentre, simulation-based comparative study using tabletop toxicology scenarios scored by triage nurses.	Thirty emergency department triage nurses from three Western Sydney hospitals (two using ATS with on-site toxicology services and one using MTS	Compared with ATS, MTS assigned consistently lower acuity triage categories and showed greater score variability, particularly for rare but highly toxic presentations that	The simulation design limits real-world generalisability, volunteer sampling may introduce bias, and differences in local toxicology support between hospitals may have

Authors, year and country	Aim	Research design	Sample, setting	Main findings	Conceptual and methodological issues
			without local toxicology support) each triaged eight standardized poisoning scenarios.	may initially appear clinically well.	confounded triage decisions rather than triage scale characteristics alone.
Leite et al. (2015), Portugal (19).	To describe an unselected ED chest pain population, characterize patients with ACS, and evaluate the prognostic value of the MTS and the HEART score for short-term risk stratification.	Single-centre retrospective observational cohort study.	233 adults presenting with non-traumatic chest pain to the emergency department of a Portuguese tertiary referral hospital.	Patients triaged as red/orange by MTS had significantly higher ACS rates, and the HEART score accurately predicted 6-week major adverse cardiac events (c-statistic 0.88), with clear discrimination between low-, intermediate- and high-risk groups.	The retrospective single-centre design limits generalisability; the HEART score was applied post hoc with incomplete data for some patients, and the use of conventional (non-high-sensitivity) troponin may have underestimated diagnostic performance.
Ingielewicz et al. (2025), Poland (13).	To directly compare the MTS and ESI in terms of classification agreement and their ability to predict critical clinical events in an emergency	Single-centre retrospective observational cohort study conducted during a six-month parallel-use transition period	1,072 consecutively triaged patients of all ages attending a full-profile emergency department in	MTS and ESI showed moderate agreement (κ = 0.51); both demonstrated a clear monotonic association between higher	Generalisability is limited by a single-centre design, partial ED coverage (~7% of visits), pandemic-era data collection, lack of an external gold

Authors, year and country	Aim	Research design	Sample, setting	Main findings	Conceptual and methodological issues
	department population.	between triage systems.	northern Poland, each assessed simultaneously with both MTS and ESI.	urgency and increased risk of critical events, but ESI concentrated nearly 80% of patients in level 3, whereas MTS produced a more balanced distribution across mid-level categories.	standard, single-rater triage, and potential learning effects during system transition.
Zaboli et al. (2025), Italy (14).	To compare the ability of the NEWS and the MTS to predict short-term adverse outcomes in emergency department patients.	Retrospective observational cohort study with comparative prognostic performance analysis.	1,555 adult patients presenting to a single Italian emergency department, triaged using MTS, with NEWS retrospectively calculated from initial vital signs recorded at presentation.	NEWS demonstrated superior discrimination for mortality, ICU admission, and clinical deterioration compared with MTS, while MTS remained useful for organisational prioritisation rather than outcome prediction.	Retrospective single-centre design, NEWS not applied in real-time triage, reliance on short-term outcomes, and conceptual overlap between physiological risk scoring and organisational triage limit generalisability and interpretability.
Speake et al. (2003), United Kingdom (18).	To assess whether nurses using the MTS can accurately identify chest pain	Prospective observational cohort study with diagnostic accuracy analysis	167 consecutive chest pain patients presenting over	Nurse-applied MTS demonstrated high sensitivity (86.8%) and moderate	The use of history-based "gold standard" rather than confirmed

Authors, year and country	Aim	Research design	Sample, setting	Main findings	Conceptual and methodological issues
	patients requiring immediate ECG and physician assessment within ten minutes.	against an evidence-based reference standard.	four weeks to the emergency department of a large UK inner-city teaching hospital.	specificity (72.4%) for identifying high-risk cardiac chest pain, indicating effective prioritisation but with some under- and over-triage.	diagnoses, short triage encounters, small single-centre sample, and potential training-related variability limit generalisability and diagnostic precision.
Ingielewicz et al. (2024), Poland (35).	To critically examine whether an ideal emergency department triage system exists and to explore future directions for improving triage accuracy and safety.	Narrative review and conceptual analysis of existing triage systems and validation approaches.	Not applicable, as the article synthesizes international evidence from studies of emergency department triage systems rather than reporting primary patient data.	No current triage system can be considered "perfect," as all demonstrate trade-offs between speed, simplicity, reliability, and prognostic accuracy, with emerging evidence suggesting that artificial intelligence and decision-support tools may enhance traditional nurse-led triage.	Conclusions are limited by narrative (non-systematic) methodology, reliance on heterogeneous prior studies, absence of empirical validation, and the inherent challenge of separating organisational triage performance from outcome prediction.
Zaboli et al. (2020), Italy (21).	To evaluate the effectiveness of the MTS in appropriately prioritising	Retrospective observational cohort study with diagnostic	492 adult patients with confirmed pulmonary embolism	The MTS correctly assigned high urgency to most patients with	Retrospective single-centre design, inclusion limited to

Authors, year and country	Aim	Research design	Sample, setting	Main findings	Conceptual and methodological issues
	emergency department patients ultimately diagnosed with pulmonary embolism presenting with dyspnoea, chest pain, or collapse.	performance analysis.	presenting to a single Italian emergency department, triaged using the Manchester Triage System.	haemodynamically unstable pulmonary embolism but frequently undertriaged clinically stable yet high-risk patients, particularly those presenting with dyspnoea alone.	confirmed pulmonary embolism cases (spectrum bias), lack of a prospective clinical reference standard at triage, and reliance on MTS discriminators not specific to thromboembolic disease limit validity and generalisability.
Azeredo et al. (2015), Brazil and Portugal (international academic collaboration) (10).	To systematically analyse the efficacy of the MTS for risk classification of patients presenting to emergency departments.	Systematic review of the literature with qualitative synthesis and classification of included studies by level of evidence.	Twenty-two primary studies published between 2002 and 2013, conducted mainly in European emergency departments (especially the Netherlands and Portugal), involving adult and paediatric ED populations as well as disease-	Most included studies supported the validity and clinical usefulness of the MTS for prioritisation, admission prediction, and short-term mortality prediction. However, clinically relevant under-triage and over-triage persisted, particularly in	Conclusions are limited by heterogeneous study designs and outcomes, the predominance of observational and descriptive evidence, variable reference standards for "severity," the exclusion of pre-2002 literature, and the lack of meta-analyses to quantify

Authors, year and country	Aim	Research design	Sample, setting	Main findings	Conceptual and methodological issues
			specific cohorts (e.g. ACS, PE, chest pain).	elderly patients, children, and those with atypical presentations.	pooled diagnostic performance.
Kuriyama et al. (2017), Japan (15).	To systematically describe and compare the reference standards and outcome measures used to assess the validity of five-level emergency department triage systems.	Systematic review conducted according to PRISMA guidelines, focusing on methodological approaches to criterion and construct validity rather than pooled performance estimates.	Fifty-seven validation studies of fourteen five-level triage systems (including MTS, ESI, CTAS, and ATS) encompass adult, paediatric, and mixed ED populations across multiple countries and settings.	Most validation studies relied on construct validity using proxies such as admission, resource use, and mortality. In contrast, true criterion validity was uncommon and inconsistently defined, with wide heterogeneity in reference standards across the same triage system.	The absence of a gold standard for "urgency," inconsistent use of reference standards, frequent conflation of urgency with severity or resource use, and lack of bias assessment limit the interpretability and comparability of triage validity research.
Blomaard et al. (2020), The Netherlands (30).	To investigate whether adding geriatric vulnerability screening (APOP) to MTS urgency levels improves the prediction of 30-day mortality in older emergency department patients.	Secondary analysis of a prospective multicentre observational cohort study (the APOP study).	2,608 consecutive patients aged ≥70 years presenting to the emergency departments of four Dutch hospitals, triaged with MTS and assessed with the APOP geriatric screening tool.	Within every MTS urgency category, patients identified as high risk by geriatric screening had approximately threefold higher 30-day mortality, and combining APOP with MTS substantially	Exclusion of immediately life-threatened (red-triage) patients, retrospective calculation of the geriatric screener, modest overall predictive performance, focus on mortality rather

Authors, year and country	Aim	Research design	Sample, setting	Main findings	Conceptual and methodological issues
				improved explained variance and discrimination for mortality compared with MTS alone.	than true urgency, and use of a Netherlands-specific screening tool limit generalisability and causal interpretation.
Zachariasse et al. (2021), a multicountry European study led from the Netherlands (with sites in the UK, Austria, and Portugal) (26).	To develop and validate age-specific vital-sign–based discriminators to improve the performance of the MTS in paediatric emergency department patients.	Prospective multicentre observational cohort study with derivation-validation split (training and test sets) embedded in the TrIAGE project.	117,438 consecutive children under 16 years presenting to five European emergency departments between 2012 and 2015, all triaged using the MTS.	Adding six new heart-rate and respiratory-rate discriminators to selected MTS flowcharts reduced undertriage. They modestly improved sensitivity for high-urgency cases (from 0.66 to 0.71), at the cost of a small increase in overtriage.	Use of a composite proxy reference standard for "true urgency," substantial missing vital-sign data requiring imputation, small absolute performance gains, lack of external validation, and the inherent limitation of modifying a flowchart-based system with physiological thresholds constrain generalisability and clinical impact.
Simon Junior H et al. (2022), Brazil (29).	To describe and discuss the main	Narrative, descriptive literature	Not applicable — the article does	The paper concludes that	The absence of a systematic

Authors, year and country	Aim	Research design	Sample, setting	Main findings	Conceptual and methodological issues
	paediatric emergency department triage systems in use worldwide, with emphasis on their structure, rationale, and clinical application rather than formal performance comparison.	review (non-systematic), without explicit search strategy, inclusion criteria, or quality appraisal.	not analyse primary data but draws on published studies and official descriptions of paediatric triage systems used in paediatric and mixed emergency departments internationally.	several paediatric triage systems (including MTS, ESI, CTAS, and ATS) are widely used and feasible. Still, that evidence supporting their validity and reliability in children is limited and inconsistent, particularly for younger age groups.	methodology, the lack of quantitative synthesis, reliance on heterogeneous secondary sources, and the conceptual difficulty in distinguishing urgency from severity in children limit the strength and generalisability of the conclusions.

Authors, year and country	Aim	Research design	Sample, setting	Main findings	Conceptual and methodological issues
Brouns et al. (2019), The Netherlands (31).	To evaluate the performance of the MTS in older emergency department patients (≥65 years) by assessing its ability to predict resource utilisation, emergency department length of stay, hospitalisation, and in-hospital mortality, and to compare its performance with that in younger	Retrospective observational cohort study with comparative predictive performance analysis.	7,108 emergency department visits by patients aged ≥65 years and 13,767 visits by patients aged 18–64 years, conducted at a single Dutch teaching hospital emergency department between 2011 and 2012.	Higher MTS urgency was associated with increased resource use, admissions, and mortality across all age groups, but predictive performance for in-hospital mortality was lower in older patients (AUC 0.71 vs 0.79), with better discrimination in older surgical patients than in older medical patients.	Retrospective single-centre design, exclusion of certain patient groups (e.g. cardiology and gynaecology), reliance on proxy outcomes rather than true urgency, lack of adjustment for comorbidity or frailty, and health-system–specific organisation limit generalisability and

Authors, year and country	Aim	Research design	Sample, setting	Main findings	Conceptual and methodological issues
	patients and between medical and surgical subgroups.				causal interpretation.
Ausserhofer et al. (2020), Italy (20).	To evaluate the predictive validity and safety of the MTS in emergency department patients presenting with dyspnoea, focusing on short-term mortality and patterns of under- and overtriage.	Retrospective observational cohort study with diagnostic performance analysis using mortality as the clinical endpoint.	4,076 adult patients presenting to a single Italian emergency department with dyspnoea between January 2017 and June 2019, all triaged using the Manchester Triage System.	High MTS priority (orange/red) showed acceptable discrimination for seven-day mortality (AUC 0.73), high sensitivity (75.9%) and negative predictive value (98.7%), indicating low undertriage but substantial overtriage in patients with dyspnoea.	Single-centre retrospective design, use of seven-day mortality as a proxy for urgency, potential triage nurse variability not directly assessed, exclusion of trauma and specific populations, and reliance on outcome-based validation limit causal inference and generalisability.
Magalhães-Barbosa et al. (2019), Brazil (28).	To systematically review and critically appraise the evidence on the inter-rater and intra-rater reliability of five-level emergency department triage systems used in paediatric patients.	Systematic review conducted according to PRISMA guidelines, including qualitative synthesis without meta-analysis due to methodological heterogeneity.	Twenty reliability studies involving paediatric patients aged 0–18 years, conducted across multiple countries and emergency department	Moderate-quality studies with actual patients showed substantial to almost perfect reliability for PedCTAS, MTS, and ESI v4 (quadratic weighted kappa generally ranging	The evidence base is weakened by the predominance of low-quality studies, frequent use of non-representative scenarios, inconsistent or poorly reported kappa weighting,

Authors, year and country	Aim	Research design	Sample, setting	Main findings	Conceptual and methodological issues
			settings, using both actual patients (n=10 studies) and hypothetical clinical scenarios (n=10 studies), covering nine different triage systems including MTS, PedCTAS, and ESI.	from 0.61 to 0.83). In contrast, most scenario-based studies demonstrated lower, more variable agreement.	convenience sampling of raters, limited blinding and independence between raters, exclusion of high-acuity patients, and strong country-of-origin bias limiting generalisability.
Castro Delgado et al. (2022), Spain (34).	To estimate and compare the sensitivity and specificity of the Spanish Prehospital Advanced Triage Method (META) and the MTS when applied to mass-casualty incident (MCI) patients, using the RTS as the reference standard.	Retrospective comparative diagnostic accuracy study applying two triage algorithms to existing MCI registry data.	134 patients of all ages involved in real mass-casualty incidents and transported to the Central University Hospital of Asturias, Spain, were identified from the Asturias UIED population-based MCI database (2017–2019).	META demonstrated substantially higher overall sensitivity (79.9% vs 30.6%) and specificity (89.9% vs 66.0%) than MTS, with markedly lower over-triage and under-triage rates, indicating superior performance for identifying severely injured patients in MCIs.	Retrospective application of triage algorithms, small sample size, limited representation of MCI types, exclusion of on-scene deaths, reliance on RTS as an imperfect gold standard, and inherent challenges of validating triage tools in low-frequency disaster settings limit external validity.

Authors, year and country	Aim	Research design	Sample, setting	Main findings	Conceptual and methodological issues
García-Azorín et al. (2023), Spain (23).	To evaluate the frequency of subtriage by the MTS in emergency department patients presenting with headache and its association with secondary headache diagnoses.	Retrospective observational cohort study.	1120 adult patients presenting to a single Spanish emergency department with headache, all triaged using the MTS, with 248 patients included.	Patients with secondary headaches were significantly more likely to be assigned to low-urgency MTS categories than those with primary headaches, indicating clinically relevant subtriage.	A single-centre, retrospective design, reliance on final diagnosis as a proxy for urgency, absence of prospective outcome validation, exclusion of paediatric patients, and restriction to a single presenting symptom limit generalisability and causal interpretation.
Nguyen-Tat et al. (2018), Germany (32).	To evaluate the performance of the MTS in risk stratification of emergency department patients presenting with suspected acute gastrointestinal bleeding.	Retrospective observational cohort study.	284 adult patients presenting with suspected gastrointestinal bleeding to a single German tertiary emergency department were identified among 5,689 patients with gastrointestinal complaints.	Higher MTS urgency levels were significantly associated with confirmed gastrointestinal bleeding and need for endoscopic intervention, with MTS showing high sensitivity but low specificity for identifying true bleeding cases.	A single-centre retrospective design, reliance on endoscopic findings as a surrogate for urgency, low specificity leading to overtriage, exclusion of prehospital data, and absence of comparison with established bleeding risk scores limit

Authors, year and country	Aim	Research design	Sample, setting	Main findings	Conceptual and methodological issues
					generalisability and clinical precision.
Grouse et al. (2009), Australia (16).	To evaluate the inter-rater reliability of the MTS when used by trained nurses in an Australian emergency department.	Single-centre inter-rater reliability study using retrospective, paper-based triage scenarios derived from real patients.	50 triage scenarios based on 50 consecutive emergency department presentations, independently assessed by 20 MTS-trained nurses at an urban Australian teaching hospital.	The MTS demonstrated fair to substantial inter-rater reliability, with weighted kappa values ranging from 0.40 to 0.80 (median ≈0.63) and an overall concurrence of 75%, indicating consistent triage categorisation among nurses.	The use of paper-based scenarios rather than live patient encounters, the single-centre design, the small number of scenarios, potential institutional training effects, and limited external validity restrict generalisability to other emergency department settings.
Brigo et al. (2022), Italy (22).	To assess the performance of the MTS in prioritising emergency department patients presenting with non-traumatic headache, with a focus on identifying urgent and severe neurological conditions.	Retrospective observational cohort study.	3,002 adult patients presenting with non-traumatic headache to a single Italian emergency department over 30 months, all triaged using the Manchester Triage System.	The MTS showed good overall discriminatory ability (AUC 0,734) and very high negative predictive value for severe neurological disease, but low specificity, with only a minority of patients with urgent neurological	A single-centre, retrospective design, reliance on final diagnosis as a proxy for urgency, low event rates of severe neurological conditions, absence of prospective outcome validation, and restriction to headache presentations limit

Authors, year and country	Aim	Research design	Sample, setting	Main findings	Conceptual and methodological issues
				conditions assigned high-urgency triage categories.	generalisability and causal interpretation.
Zaboli et al. (2022), Italy (24).	To assess the performance of the MTS in the initial prioritisation of emergency department patients presenting with TLOC.	Retrospective observational cohort study.	2,291 adult patients presenting with transitory loss of consciousness to a single Italian emergency department, all triaged using the Manchester Triage System.	Higher MTS urgency levels were associated with greater rates of hospital admission and adverse clinical outcomes. Still, a clinically relevant proportion of patients with serious underlying causes of TLOC were initially assigned lower urgency categories, indicating undertriage.	Single-centre retrospective design, reliance on outcome- and diagnosis-based proxies rather than real-time urgency, heterogeneity of TLOC aetiologies, lack of prospective validation, and absence of comparison with dedicated syncope risk scores limit generalisability and interpretability.
Hinson et al. (2019), international (predominantly North America and Europe) (12).	To systematically evaluate the performance of emergency department triage systems in predicting clinical outcomes, including mortality, critical illness, and hospital admission.	Systematic review conducted according to PRISMA guidelines.	182 eligible studies of adult emergency department triage systems worldwide were identified, of which 50 provided sufficient data for comparative	Across triage systems, sensitivity was generally high for emergency department mortality but substantially lower for critical illness and post-emergency outcomes, with	Marked heterogeneity in study designs, outcomes, and reference standards, heavy reliance on proxy endpoints rather than true urgency, inconsistent reporting of

Authors, year and country	Aim	Research design	Sample, setting	Main findings	Conceptual and methodological issues
			performance analysis across systems, including ATS, CTAS, ESI, MTS, and SATS.	frequent undertriage of patients requiring hospitalisation and only moderate, variable inter-rater reliability.	reliability metrics, and lack of a gold standard for triage validity limit comparability and the strength of conclusions.
Aeimchanbanjong et al. (2017), Thailand (27).	To compare the reliability and validity of five pediatric emergency department triage systems (MTS, ESI v4, CTAS, ATS, and Ramathibodi Triage System) in identifying patient acuity and predicting hospital admission.	Prospective, single-centre observational study conducted in two phases assessing inter-rater reliability and predictive validity.	A total of 1,041 pediatric patients (<15 years) presenting to a tertiary university hospital emergency department in Bangkok, Thailand, between April and July 2015 were included after exclusions.	RTS showed almost perfect inter-rater reliability (κ=1.0), while ESI demonstrated the best validity for predicting hospital admission (AUC 0.78), with MTS showing moderate reliability and moderate predictive performance (AUC 0.70).	Validity was assessed using hospital admission as a proxy for urgency due to the lack of a gold standard; the single-centre design limits generalizability, and moderate sensitivity across systems indicates a persistent risk of undertriage in pediatric emergency care.
Zachariasse et al. (2017), the Netherlands (11).	To evaluate the validity of the MTS in emergency care by examining its association with markers of clinical	Prospective observational cohort study.	288,663 consecutive emergency department patients presenting to a large Dutch	Increasing MTS urgency levels were strongly associated with higher rates of hospital admission, diagnostic and therapeutic	Reliance on proxy outcomes rather than a true gold standard for urgency, observational design, limited

Authors, year and country	Aim	Research design	Sample, setting	Main findings	Conceptual and methodological issues
	urgency and patient outcomes.		teaching hospital and its affiliated emergency care network, all prospectively triaged using the Manchester Triage System.	resource use, intensive care admission, and short-term mortality, supporting construct validity but revealing overlap between urgency categories.	assessment of downstream consequences of mistriage, and absence of direct comparison with alternative triage systems constrain interpretability despite the large sample size.
Gräff et al. (2019), Germany (17).	To evaluate the validity of the MTS in identifying emergency department patients who require immediate life-saving interventions (LSI) or acute emergency treatment (AET) upon arrival.	Prospective single-centre observational cohort study with real-time external observation of interventions.	1,762 adult patients presenting to the emergency department of the University Hospital Bonn, Germany, over four months (October 2014–February 2015), all triaged using the Manchester Triage System.	MTS demonstrated high negative predictive value and accuracy for identifying critically ill patients, with sensitivities of 75.0% for life-saving interventions and 82.0% for acute emergency treatments. In contrast, no patients requiring LSI were triaged to low-urgency categories (green or blue).	Single-centre design, short observation period, low absolute number of LSI cases, potential observer effect, reliance on expert-defined intervention categories rather than a universal gold standard, and limited assessment of overtriage restrict generalisability despite the prospective methodology.

Authors, year and country	Aim	Research design	Sample, setting	Main findings	Conceptual and methodological issues
Gräff et al. (2017), Germany (25).	To assess the validity of the MTS in adequately prioritising emergency department patients presenting with sepsis, severe sepsis, or sepsis with circulatory dysfunction at first triage.	Single-centre retrospective observational cohort study using pseudo-random sampling of ED visits and retrospective sepsis classification based on SIRS criteria.	20,836 adult patients (>16 years) presenting to the emergency department of the University Hospital Bonn, Germany, between July 2012 and March 2014, of whom 801 patients (3.8%) fulfilled criteria for sepsis.	MTS correctly prioritised 70.4% of patients with sepsis, 84.5% with severe sepsis, and only 61.5% with sepsis and circulatory dysfunction, with approximately 30% of septic patients undertriaged to low-acuity categories, indicating moderate sensitivity and systematic undertriage.	Retrospective identification of sepsis, reliance on SIRS rather than Sepsis-3 definitions, single-centre design, pseudo-random sampling, lack of a sepsis-specific MTS flowchart, and misalignment between MTS discriminators and sepsis vital-sign thresholds limit generalisability and highlight structural limitations of MTS for septic patients.

Legend: ACS – Acute Coronary Syndrome; APOP – Acute Presenting Older Patient (geriatric screening tool); ATS – Australasian Triage Scale; AUC – Area Under the Curve; AET – Acute Emergency Treatment; CI – Confidence Interval; CTAS – Canadian Triage and Acuity Scale; ED – Emergency Department; ESI – Emergency Severity Index; GI – Gastrointestinal; HEART score – History, ECG, Age, Risk factors, Troponin score; ICU – Intensive Care Unit; IQR – Interquartile Range; κ (kappa) – Cohen's kappa coefficient; LMICs – Low- and Middle-Income Countries; LOS – Length of Stay; LSI – Life-Saving Intervention; META – Método Español de Triaje Avanzado (Spanish Prehospital Advanced Triage Method); MCI – Mass-Casualty Incident; MTS – Manchester Triage System; NEWS – National Early Warning Score; NPV – Negative Predictive Value; OR – Odds Ratio; PE – Pulmonary Embolism; PEWS – Paediatric Early Warning Score; PPV – Positive Predictive Value; PRISMA – Preferred Reporting Items for Systematic Reviews and Meta-Analyses; ROC – Receiver Operating Characteristic; RTS – Revised Trauma Score; SATS – South African Triage Scale; SD – Standard Deviation; SIRS – Systemic Inflammatory Response Syndrome; TLOC – Transitory Loss of Consciousness; UK – United Kingdom
