# Supplementary material for: Manchester Triage System: Effectiveness, Limitations, and Global Implementation—A Structured Narrative Review
Source: Healthcare (Basel). 2026 Aug 12;14(16):2502. doi: 10.3390/healthcare14162502 (PMC13512649; doi:10.3390/healthcare14162502)
Supplement: Supplementary file 1 [file healthcare-14-02502-s001.zip › healthcare-4457068-Table S2.pdf]

Supplementary Table S2. Role of all references used in the narrative review

Ref. number	Publication category	Role in the manuscript	Section(s)	Core publications
1	Narrative overview	Provides a global overview of emergency triage and establishes the broader context for MTS.	Introduction	No
2	Retrospective observational cohort study	Supports the association of MTS categories with hospital admission and mortality.	Introduction	No
3	MTS textbook/manual	Defines the official MTS structure, flowcharts, discriminators, urgency categories, and waiting-time targets.	Introduction; Background and development of MTS; Core structure and triage flowcharts	No
4	Simulation-based reliability and validity study	Provides evidence on MTS reliability and validity and supports discussion of inter-rater variability.	Introduction; Background and development of MTS; Validation and reliability studies; Critiques and limitations; Comparison with alternative systems	No
5	Systematic review	Provides contextual synthesis of previous evidence on MTS validity, reliability, undertriage, and overtriage; not treated as independent empirical evidence.	Introduction; Validation and reliability studies; Critiques and limitations; Comparison with previous reviews	Yes
6	Methodological guidance for narrative reviews	Supports the structured narrative-review design and flexible interpretive synthesis.	Methods	No
7	Methodological guidance for narrative reviews	Provides methodological guidance for transparent narrative biomedical reviews.	Methods	No
8	Narrative review quality-assessment tool	Supports quality and reporting principles for narrative reviews (SANRA).	Methods	No
9	Thematic analysis methodology	Provides the methodological framework for inductive thematic analysis and coding.	Methods	No
33	Simulation-based comparative study	Compares MTS with ATS in poisoned patients and supports the alternative-system comparison.	Comparison with alternative systems	Yes
19	Retrospective observational cohort study	Evaluates MTS and HEART score performance in chest-pain patients.	Clinical effectiveness in practice; International adaptations; Critiques and limitations	Yes
13	Retrospective comparative cohort study	Directly compares MTS with ESI for classification and prediction of critical events.	Comparison with alternative systems; Comparison with previous reviews	Yes
14	Retrospective comparative prognostic study	Compares MTS with NEWS across mortality, admission, ICU, and other outcomes.	Validation and reliability studies; Clinical effectiveness in practice; Comparison with previous reviews	Yes
18	Prospective observational study	Evaluates MTS identification of high-risk chest-pain patients requiring rapid assessment.	Clinical effectiveness in practice	Yes
35	Narrative review	Provides a conceptual comparison of major triage systems and future improvement strategies.	Comparison with alternative systems; Comparison with previous reviews	Yes
21	Retrospective observational cohort study	Evaluates MTS prioritisation in patients with pulmonary embolism.	Validation and reliability studies; Clinical effectiveness in practice	Yes
10	Systematic review	Synthesises evidence on MTS efficacy, validity, and clinical usefulness.	International adaptations; Critiques and limitations; Comparison with previous reviews	Yes
15	Systematic review	Examines reference standards and outcome measures used to validate five-level triage systems.	Validation and reliability studies; Comparison with alternative systems; Comparison with previous reviews	Yes
30	Secondary analysis of a prospective multicentre cohort	Evaluates the added prognostic value of geriatric screening alongside MTS urgency.	Validation and reliability studies; International adaptations; Comparison with alternative systems; Comparison with previous reviews	Yes
26	Prospective multicentre observational cohort study	Assesses age-specific vital-sign discriminators for improving paediatric MTS prioritisation.	Background and development of MTS; Core structure and triage flowcharts; Validation and reliability studies; Comparison with previous reviews	Yes
29	Narrative review	Provides contextual comparison of paediatric triage systems and their limitations.	Validation and reliability studies; Comparison with alternative systems; Comparison with previous reviews	Yes
31	Retrospective observational cohort study	Evaluates MTS performance in older patients and age-related differences in predictive validity.	Core structure and triage flowcharts; Validation and reliability studies; International adaptations; Comparison with previous reviews	Yes
20	Retrospective observational cohort study	Evaluates MTS performance and mortality prediction in patients with dyspnoea.	Clinical effectiveness in practice; International adaptations	Yes
28	Systematic review	Synthesises evidence on reliability of paediatric five-level triage systems.	Validation and reliability studies; Comparison with alternative systems; Comparison with previous reviews	Yes
34	Retrospective comparative diagnostic-accuracy study	Compares MTS with the META method in mass-casualty triage.	International adaptations; Critiques and limitations	Yes
23	Retrospective observational cohort study	Evaluates MTS subtriage in patients presenting with headache.	Validation and reliability studies; Clinical effectiveness in practice; International adaptations	Yes
32	Retrospective observational cohort study	Evaluates MTS risk stratification in acute gastrointestinal bleeding.	Clinical effectiveness in practice	Yes
16	Scenario-based inter-rater reliability study	Provides inter-rater reliability evidence from MTS-trained nurses.	Validation and reliability studies; Comparison with alternative systems	Yes

Ref. number	Publication category	Role in the manuscript	Section(s)	Core publications
22	Retrospective observational cohort study	Evaluates MTS clinical-risk prioritisation in non-traumatic headache.	Validation and reliability studies; Clinical effectiveness in practice	Yes
24	Retrospective observational cohort study	Evaluates MTS assessment of transient loss of consciousness and undertriage risk.	Validation and reliability studies; Clinical effectiveness in practice	Yes
12	Systematic review	Provides a broad systematic comparison of emergency triage-system performance and supports contextual comparison with previous literature; not treated as independent empirical evidence.	Comparison with alternative systems; Comparison with previous reviews	Yes
27	Prospective validation study	Compares validity and reliability of five paediatric triage systems.	Validation and reliability studies; Critiques and limitations; Comparison with alternative systems; Comparison with previous reviews	Yes
11	Prospective observational cohort study	Provides large-scale prospective evidence for MTS construct validity and outcome associations.	Validation and reliability studies; International adaptations; Critiques and limitations; Comparison with previous reviews	Yes
17	Prospective observational cohort study	Evaluates MTS identification of patients requiring life-saving intervention or acute treatment.	Validation and reliability studies; International adaptations; Comparison with alternative systems; Comparison with previous reviews	Yes
25	Retrospective observational cohort study	Evaluates MTS validity and undertriage in patients with sepsis.	Validation and reliability studies; Clinical effectiveness in practice; International adaptations; Comparison with previous reviews	Yes
36	Narrative historical overview	Provides historical context on the evolution of triage systems.	Background and development of MTS	No
37	Cross-sectional agreement study	Supports discussion of disagreement in clinicians' assessment of urgent care needs.	Background and development of MTS	No
38	Prospective comparative triage study	Compares informal triage, ESI, and MTS in prioritising emergency patients.	Background and development of MTS; Comparison with alternative systems	No
39	Systematic review	Synthesises evidence on MTS effects on time to treatment and clinical workflow.	Background and development of MTS; Clinical effectiveness in practice; Critiques and limitations	No
40	National survey	Provides background on national emergency department triage practice.	Background and development of MTS	No
41	Narrative review	Provides contextual evidence on paediatric triage-system reliability and validity.	Core structure and triage flowcharts	No
42	Systematic review and meta-analysis	Provides pooled comparative evidence on performance of major emergency triage systems.	Core structure and triage flowcharts; Comparison with alternative systems	No
43	Narrative clinical review	Supports discussion of geriatric assessment, cognitive impairment, and vulnerability at triage.	Validation and reliability studies	No
44	External validation study	Provides external validation evidence for the APOP geriatric screening tool.	Validation and reliability studies	No
45	Prospective comparative validation study	Provides evidence on performance of local and international triage tools in children under five in Tanzania.	Validation and reliability studies; Comparison with previous reviews	No
46	Prospective observational cohort study	Provides prospective evidence on MTS validity in paediatric emergency care.	Validation and reliability studies	No
47	Repeatability/reliability study	Provides evidence on repeatability of MTS classifications in children.	Validation and reliability studies; Comparison with alternative systems	No
48	Observational comparative study	Supports the influence of nursing education and specialised training on triage accuracy.	Validation and reliability studies	No
49	Inter- and intra-observer reliability study	Provides inter- and intra-observer agreement data for MTS.	Validation and reliability studies; International adaptations; Critiques and limitations	No
50	Inter-rater reliability and accuracy study	Provides evidence on MTS inter-rater reliability and accuracy in Sweden.	Clinical effectiveness in practice; Critiques and limitations	No
51	Diagnostic-accuracy study	Evaluates sensitivity and specificity of MTS for acute coronary syndrome.	Clinical effectiveness in practice	No
52	Narrative clinical review	Provides clinical context for emergency headache assessment and management.	Clinical effectiveness in practice	No
53	Clinical review / red-flag framework	Provides the SNNOOP10 red- and orange-flag framework for secondary headache.	Clinical effectiveness in practice	No
54	Clinical practice guideline	Provides guideline-based context for syncope diagnosis and risk assessment.	Clinical effectiveness in practice	No
55	International consensus definition	Provides consensus definitions used to contextualise sepsis-related findings.	Clinical effectiveness in practice	No
56	Observational validation study	Evaluates MTS performance in patients with acute coronary syndrome.	International adaptations	No
57	Systematic review	Provides an overview of modern triage principles and comparisons among triage approaches.	International adaptations; Comparison with alternative systems	No
58	Narrative review	Provides international and organisational context for emergency department triage.	International adaptations	No
59	Observational evaluation study	Provides implementation evidence from a Brazilian emergency hospital service.	International adaptations; Critiques and limitations	No
60	Meta-analysis	Provides pooled evidence on MTS reliability and sources of heterogeneity.	Critiques and limitations	No
61	Diagnostic-accuracy study	Evaluates MTS accuracy in an emergency service.	Critiques and limitations	No
62	Integrative literature review	Synthesises literature on MTS reliability and validity for the limitations discussion.	Critiques and limitations	No
63	Narrative review	Provides context on emergency department overcrowding, consequences, and operational limitations.	Critiques and limitations	No
64	Comparative triage study	Supports comparison between five-level and three-level triage systems.	Comparison with alternative systems	No
68	Validation and reliability study	Provides validation and reliability evidence for ESI in a Spanish hospital.	Comparison with alternative systems	No
69	Comparative observational study	Directly compares ESI and MTS in trauma patients.	Comparison with alternative systems	No
66	Before-and-after implementation study	Evaluates the effect of electronic triage decision support on consistency of triage scores.	Comparison with alternative systems	No
67	Clinical guideline	Defines current CTAS guidance for comparison with MTS.	Comparison with alternative systems	No
70	Validation and reliability study	Provides validity and reliability evidence for SATS in prehospital providers.	Comparison with alternative systems	No
71	Reliability study	Provides reliability evidence for the adult SATS.	Comparison with alternative systems	No
65	Systematic review	Provides a systematic overview of primary emergency department triage assessment.	Comparison with alternative systems	No
72	Diagnostic-accuracy study	Supports discussion of point-of-care troponin decision aids as complementary future strategies; not used as direct evidence of MTS performance.	Future directions	No
73	Diagnostic-accuracy study	Supports discussion of complementary point-of-care chest-pain assessment strategies,	Future directions	No

Ref. number	Publication category	Role in the manuscript	Section(s)	Core publications
		rather than direct evaluation of MTS.		
74	Legal and ethical commentary	Provides legal and ethical context for artificial intelligence in clinical triage.	Future directions	No
75	Machine-learning prognostic study	Provides empirical evidence for machine-learning prediction using emergency triage data.	Future directions	No
76	Qualitative stakeholder study	Provides stakeholder perspectives on digitalisation of emergency-department triage.	Future directions	No
77	Scoping review	Maps current applications and evidence for artificial intelligence in hospital ED triage.	Future directions	No
78	Narrative review	Synthesises benefits, challenges, and future directions of AI-driven emergency triage.	Future directions	No

Note: "Yes" indicates inclusion among the 27 core publications subjected to inductive thematic analysis. "No" indicates use for methodological guidance, background, contextualisation, comparison, or interpretation; these references were not treated as independent core evidence.
